# Is there still a role for cytotoxic chemotherapy after targeted therapy and immunotherapy in metastatic melanoma? A case report and literature review

**DOI:** 10.1186/s40880-017-0179-6

**Published:** 2017-01-13

**Authors:** Aurélien Simon, Hampig Raphael Kourie, Joseph Kerger

**Affiliations:** Jules Bordet Institute, Free University of Brussels, Brussels, Belgium

**Keywords:** Metastatic melanoma, Chemotherapy, Immunotherapy, Checkpoint inhibitors, Vemurafenib, ATM mutation, Chemosensitivity

## Abstract

Metastatic melanoma has long been considered to have a very poor prognosis and to be chemo-resistant. However, a subgroup of patients with metastatic melanoma presents remarkable responses to chemotherapeutic agents, even in the absence of a response to modern targeted therapies and immunotherapies; accordingly, determining predictive biomarkers of the response to chemotherapies for metastatic melanoma remains a priority to guide treatment in these patients. We report a case study of a patient with B-Raf proto-oncogene serine/threonine kinase-mutated metastatic melanoma harbouring many genetic mutations. The patient did not respond to prior targeted therapies or immunotherapies but experienced a dramatic objective radiological and clinical response to subsequent dacarbazine-based chemotherapy. In the era of targeted therapies and immunotherapies for metastatic melanoma, cytotoxic chemotherapies may still represent an interesting therapeutic weapon in a well-defined subgroup of patients presenting with specific genetic and molecular features.

## Background

Malignant melanoma is a malignancy with a fast growing incidence [1, 2]. Metastatic melanoma has long been considered to exhibit a dismal prognosis and to be chemo-resistant.

In the recent era of emergent targeted therapies and immunotherapies, metastatic melanoma is the first solid tumor to benefit from this therapeutic revolution and has become the pioneer malignancy in these therapeutic areas. The presence of the B-Raf proto-oncogene serine/threonine kinase (*BRAF*) V600 mutation in 40%–50% of melanomas and its role as a predictive factor of response to BRAF inhibitors in combination with mitogen-activated protein kinase kinase (MEK) inhibitors were crucial in establishing an appropriate therapeutic management algorithm for metastatic melanomas [3].

Although melanoma has long been considered to be chemo-resistant, cytotoxic chemotherapy represented the only available therapeutic option for metastatic melanoma before the era of targeted therapies and immunotherapies. Many chemotherapy regimens only induced modest response rates; the most common regimens were dacarbazine-based and induced objective response rates (ORRs) ranging from 15% to 20% [4]. An observational study has indicated prolonged remission for 7 years [5]. The combination of dacarbazine with other agents, especially cisplatin, produced better results than dacarbazine alone in terms of ORR and progression-free survival but not overall survival [6].

Currently, in *BRAF* V600-mutated metastatic melanoma, the combination of BRAF and MEK inhibitors is considered the standard of care, with response rates exceeding 70% for first-line treatment [7]. In *BRAF* non-mutated metastatic melanoma, immune checkpoint inhibitors have been the standard of care since the approval of ipilimumab in March 2011 [8], pembrolizumab in September 2014 [9], and nivolumab in December 2014 as first-line therapies [10]. More recently, the combination of nivolumab and ipilimumab (October 2015) has shown an ORR exceeding 75%, a gain accompanied by higher and more pronounced toxicities than those observed in single-agent immunotherapy trials [11].

In this paper, we report a case of a patient with *BRAF*-mutated metastatic melanoma harbouring many genetic mutations who did not respond to targeted therapies (BRAF and MEK inhibitors) or to immune checkpoint inhibitors, such as ipilimumab and nivolumab, but presented an impressive and dramatic response to subsequent cytotoxic chemotherapy consisting of dacarbazine and cisplatin. We also discuss the potential role of chemotherapy after BRAF and MEK inhibitor treatment and immunotherapy as well as the potential interest and benefit of chemotherapy in particular subgroups of patients.

## Case report

A 56-year-old man with a history of hypercholesterolemia and myocardial infarction presented in December 2013 with a dermatologic lesion in the left lumbar region. The pathologic examination of the excisional biopsy revealed an ulcerated malignant melanoma of 6.5 mm in thickness (Breslow). The type was a superficial spreading melanoma, and the Clark level was 4.

After a wide excision of the lesion with 2 cm margins, the pathologic results of the sentinel lymph nodes showed an invasion of malignant melanoma, requiring a subsequent complete left inguinal lymph node dissection. The pathologic TNM stage was pT4bpN1acM0 according to the 7th edition of the American Joint Committee on Cancer/Union for International Cancer Control (AJCC/UICC) staging system. The primary tumor exhibited the typical *BRAF* V600E mutation.

Four months later, in April 2014, the patient presented a locoregional cutaneous and subcutaneous relapse in the lumbar region. First-line treatment consisted of the single-agent BRAF inhibitor vemurafenib, which had to be stopped, despite a clinical response, due to unacceptable toxicities, such as a grade 4 skin rash and a grade 2 daily fever. A shift to dabrafenib in combination with trametinib in a medical need programme was initiated in July 2014 and stopped in December 2014 after clinical progression of the lumbar local relapse and of multiple in-transit metastases.

Between January and March 2015, the patient received 4 injections of ipilimumab, a monoclonal anti-cytotoxic T-lymphocyte-associated protein 4 (CTLA4) antibody. The main adverse effect after the fourth injection was excessive fatigue, which was attributed to auto-immune hypophysitis with adrenal and gonadal insufficiencies requiring hormonal substitution of hydrocortisone and topic testosterone, respectively. After 4 doses of ipilimumab, positron emission tomography/computed tomography (PET/CT) unfortunately showed progressive disease and the appearance of lung and lymph node metastases.

**Fig. 1:**
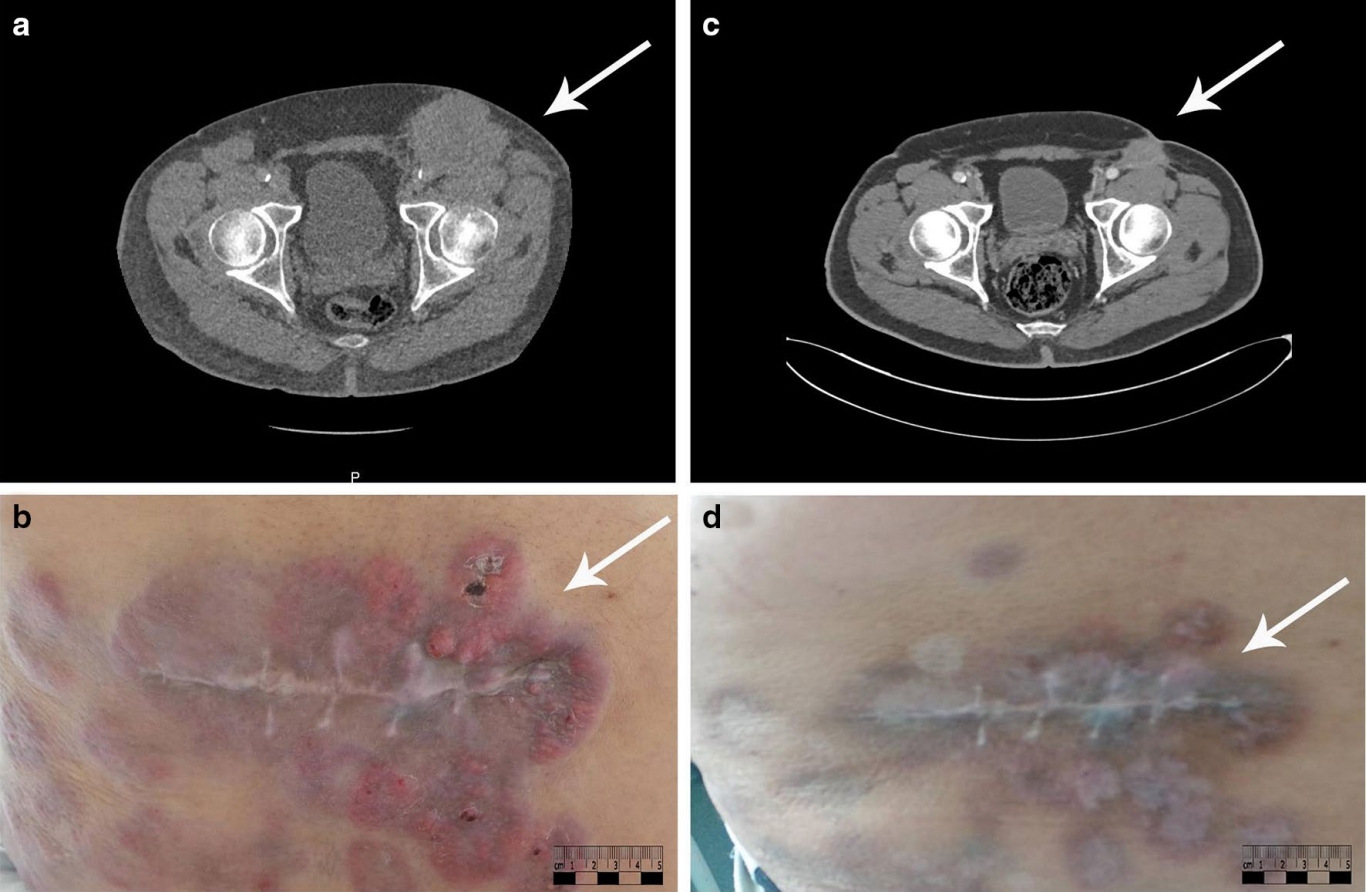
Computed tomography (CT) and macroscopic images of the inguinal lesion before and after 3 cycles of chemotherapy**. a** CT scan shows a subcutaneous metastatic melanoma lesion (*arrow*) of 76 mm × 63 mm in the left inguinal area before chemotherapy. **b** Cutaneous metastatic melanoma lesions (*arrow*) were nodular and inflammatory before chemotherapy. **c** CT scan shows that the size of the subcutaneous metastatic melanoma lesion (*arrow*) decreased to 31 mm × 35 mm, with a reduction of 48%, after 3 cycles of chemotherapy. **d** Cutaneous metastatic melanoma lesions (*arrow*) exhibited massive shrinkage, leaving a fibrotic quality of the skin, after 3 cycles of chemotherapy

Starting in July 2015, the patient was treated with nivolumab (twice every week), a monoclonal anti-programmed cell death 1 (PD-1) antibody, within the framework of a phase II trial. A CT scan performed after 8 weeks of nivolumab treatment demonstrated clear disease progression, including cutaneous and subcutaneous, lymph node, pleuro-pulmonary, renal, and peritoneal metastases (Fig. 1a, b). At this point, biological analyses indicated elevated serum lactate dehydrogenase (LDH) levels.

Two molecular analyses of the tumor, one using OncoDeep (OncoDNA, Gosselies, Belgium) and the other using the TruSeq Illumina Cancer Panel (Illumina Inc., San Diego, CA, USA), were performed after the failure of nivolumab (at the end of August 2015). The results were discordant: the OncoDNA detected only one *BRAF* V600E mutation, whereas the Illumina Panel (TruSeq Amplicon Cancer Panel) detected *BRAF* V600E-F-box and WD repeat domain containing 7 R385C mutations (*FBXW7*), a kinase domain insert receptor Q472H variant (*KDR*), a V-Ki-ras2 Kirsten rat sarcoma viral oncogene homologue G12D mutation (*KRAS*), a tumor protein P53 P72R variant (*P53*), and a polymorphism of Ataxia telangiectasia mutated (*ATM*) −c.8850 + 60A > G.

Since September 2015, the patient had received 4 cycles of cytotoxic chemotherapy consisting of intravenous injections of dacarbazine (350 mg/m^2^) and cisplatin (25 mg/m^2^) for 3 consecutive days, given every 3–4 weeks. An ongoing, impressive, and dramatic response of all metastases (the sizes decreased by more than 80%) was documented after 3 cycles of chemotherapy (Fig. 1c, d).

During chemotherapy, a second biopsy was performed, and the same mutations were detected, but there was a difference in the percentage of cells with the *BRAF* V600E mutation (41% in August 2015 and 36% in November 2015).

After the failure of checkpoint inhibitors, an immunological biomarker and microenvironment analysis revealed the absence of PD-1/programmed death-ligand 1 (PD-L1) (Ventana biomarker assay) staining, the absence of CD20 (B cells) staining, and diffuse and weak CD3 (T cells) staining.

**Fig. 2:**
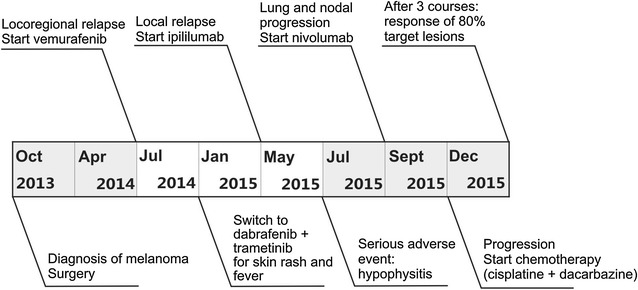
Flow chart summarizing the treatment provided to this patient

We summarized the treatment provided to this patient in a flow chart (Fig. 2).

## Discussion

The particular clinical feature of our case was the presence of multiple genetic mutations in the tumor, which did not respond to targeted therapies or checkpoint inhibitors but exhibited a major response to dacarbazine and cisplatin combination chemotherapy in fifth-line therapy.

Apart from the differences (e.g., depth of coverage, number of genes analyzed, and devices and analysis systems) between the OncoDeep test and Illumina panel, the discordant results (i.e., the greater number of mutations detected using the Illumina panel) may be explained by tumor heterogeneity due to the different origins of the two samples.

This rare case raises a number of questions. Is there a subgroup of metastatic melanomas that still benefit from cytotoxic chemotherapy? Are there any predictive factors leading to this response? Should the presence of the observed genetic mutations in metastatic melanoma be considered a predictive factor for chemo-sensitivity? Is there a potential role for immune checkpoint inhibitors that render these tumors more chemo-sensitive by modifying the microenvironment?

Many hypotheses can be considered with respect to these questions. The first and strongest hypothesis is that the observed response is explained by the presence of an *ATM* mutation in this tumor. The *ATM* gene is responsible for the repair of DNA double-strand breaks [12]. The presence of an *ATM* mutation leads to a dysfunction in the repair process for DNA double-strand breaks and consequently could render the tumor more chemo-sensitive, especially to platinum agents, according to the literature [13, 14]. This process is comparable to breast cancer 1 gene (*BRCA1*)-mutated breast cancer, which exhibits acceptable sensitivity to platinum agents and/or poly(ADP-ribose) polymerase (PARP) inhibitors. By extrapolation, the use of PARP inhibitors could be considered an interesting therapeutic modality in the progression of chemotherapy.

A second hypothesis may be the “terra incognita” effect of immunotherapy (anti-CTLA4 and anti-PD-1) on the subsequent response to chemotherapy. Cytotoxic agents seem to enhance the anti-tumor immune response by releasing antigens after cellular destruction [15, 16]. Some basic researches on immunological biomarkers and microenvironments, e.g., studies of intratumoral lymphoid infiltrates with intratumoral PD-L1 expression and the interferon-gamma pathway in tumor tissue, show that these factors can predict the response to immune checkpoint inhibitors [17, 18]. In fact, high expression of PD-L1 and the presence of tumor-infiltrating lymphocytes are associated with better responses to checkpoint inhibitors [19, 20].

Finally, to answer these questions, a retrospective mutation-based study could be used to evaluate the response rate of metastatic melanomas to different therapeutic modalities according to each individual mutation. Based on the results of this observational study, a randomized trial aimed at comparing chemotherapy to targeted therapies and checkpoint inhibitors based on different mutation profiles should be launched. A similar methodology will be used to confirm or clarify the sustained role of chemotherapy in well-defined subgroups of patients, despite the encouraging and promising results of targeted therapies and/or immunotherapies.

## Conclusions

We are currently in an exciting era of promising new treatment options for malignant melanoma. Cytotoxic chemotherapy (especially dacarbazine and cisplatin) could nevertheless remain an invaluable therapeutic weapon in specific cases with chemosensitizing mutations.
